# MTFR2 promotes endometrial carcinoma cell proliferation and growth via the miR-132-3p/PI3K/Akt signaling pathway

**DOI:** 10.3389/fmed.2024.1505071

**Published:** 2025-03-10

**Authors:** Zhijun Niu, Yue Zhang, Yishan Wang, Dongxia Liu, Junmin Wang, Tingting Shi, Xia Xu, Lei Li

**Affiliations:** Department of Obstetrics and Gynecology, The Third Affiliated Hospital of Zhengzhou University, Zhengzhou, China

**Keywords:** MTFR2, endometrial cancer, miR-132-3p, PI3K/AKT, signaling pathway

## Abstract

**Objective:**

Understanding the mechanisms underlying endometrial cancer progression is crucial for the development of effective targeted therapies. In this study, we investigated the role of MTFR2 in endometrial cancer cell.

**Methods:**

The expression of MTFR2 in endometrial cancer was analyzed using The Cancer Genome Atlas (TCGA) dataset and detected in endometrial cancer tissues and cells, respectively. Gain-of-function and loss-of-function approaches were utilized to investigate the impact of MTFR2 on endometrial cancer cell proliferation and tumorigenesis in both *in vitro* and *in vivo* settings. Computational tools were employed to predict microRNAs (miRNAs) that potentially regulate MTFR2, and these predictions were experimentally validated.

**Results:**

The expression of MTFR2 is enhanced in endometrial carcinoma, and it is positively correlated with the poor prognosis of patients. Functional studies show that MTFR2 promoted the proliferation, migration and invasion of endometrial cancer cells. Bioinformatics analysis and luciferase assays identified that MTFR2 is a potential target of miR-132-3p, and transfection with miR-132-3p mimics attenuated the MTFR2-induced activation of the PI3K/Akt pathway.

**Conclusion:**

Our findings highlight the critical role of MTFR2 in promoting endometrial cancer cell proliferation and growth through the miR-132-3p/PI3K/Akt signaling pathway. Targeting this signaling axis may offer potential therapeutic strategies for endometrial cancer treatment.

## 1 Introduction

Endometrial carcinoma (EC) is a prevalent gynecological malignancy characterized by abnormal growth of the endometrial lining. It is the fourth most common cancer among women worldwide, with an increasing incidence in recent years (1–3). Despite advances in diagnosis and treatment, the prognosis for advanced-stage endometrial cancer remains poor, highlighting the need for a deeper understanding of the molecular mechanisms driving its progression.

Mitochondrial fission regulator 2 (MTFR2) is a mitochondrial protein that plays a critical role in regulating mitochondrial dynamics and function (4, 5). It has been implicated in various cellular processes, including metabolism, apoptosis, and cell proliferation. Recent studies have shed light on the involvement of MTFR2 in tumorigenesis (6). For instance, a relevant study has demonstrated that MTFR2 is upregulated and promotes breast cancer cell growth and metastasis (7). Additionally, another research also found the expression of MTFR2 is significantly associated with clinicopathologic characteristics and prognosis of esophageal squamous cell carcinoma (8). These findings suggest that MTFR2 may have a broader role in promoting tumor progression beyond pancreatic and liver cancers.

MicroRNAs (miRNAs) are small non-coding RNA molecules that play crucial roles in post-transcriptional regulation of gene expression by binding to the 3′ untranslated regions (UTRs) of target messenger RNAs (mRNAs), leading to their degradation or translational repression. They are involved in various physiological and pathological processes, including cancer development and progression (9, 10). In the context of endometrial cancer, miRNAs have emerged as key regulators of multiple signaling pathways, influencing tumor initiation, progression, metastasis, and chemoresistance. Recently, several reviews have highlighted the complex interplay between miRNAs and their targets in endometrial cancer, with studies indicating that miRNAs such as miR-21, miR-155, and miR-200 have significant implications for the diagnosis, prognosis, and therapeutic response in this malignancy (11–14). These findings underscore the importance of miRNAs as both biomarkers and therapeutic targets, offering new insights into the molecular mechanisms driving endometrial cancer. Regarding miR-132-3p, a specific miRNA, has been shown to have tumor-suppressive properties in several cancer types. Many studies have shown that miR-132-3p plays an important role in various physiological and pathological processes, particularly in the nervous system, immune response, cardiovascular diseases, and cancer (15). Researchers such as Ma have stated that miR-132-3p plays a key role in neural development and neuroplasticity, especially in influencing neuron growth, synapse formation, and plasticity, and it has potential therapeutic significance in neurodegenerative diseases, brain injury, and related neurological disorders (16). In the field of cancer, miR-132-3p has also been found to be associated with tumorigenesis and progression. It may affect tumor growth and metastasis by regulating signaling pathways involved in tumor cell proliferation, migration, and invasion (17). However, the specific mechanisms of miR-132-3p and its functions in different types of cancer are still under further investigation.

The objective of this study was to investigate the role of MTFR2 in endometrial cancer. The findings revealed elevated MTFR2 expression in endometrial cancer, which subsequently facilitated tumor cell proliferation via the activation of the phosphatidylinositol-4, 5-bisphosphate 3-kinase (PI3K)-protein kinase B (Akt) signaling pathway. We propose that this abnormal upregulation of MTFR2 is partly influenced by the posttranscriptional regulation mediated by the tumor suppressor miR-132-3p. Additionally, our analysis of clinical correlations and survival predictions indicated that MTFR2 could serve as a reliable prognostic marker for endometrial cancer diagnosis. Overall, the findings will contribute to the growing body of knowledge on EC pathogenesis and may have implications for the development of targeted therapies for this aggressive gynecological malignancy.

## 2 Materials and methods

### 2.1 Data collection and analysis

Mitochondrial fission regulator 2 expression levels of TCGA pan-cancer were downloaded from the UCSC Xena database [https://xena.ucsc.edu (V2). Accessed on: 2023.10.21]. Use The Cancer Genome Atlas (TCGA) and the Gene Expression Omnibus database (GEO) to compare the expression levels of MTFR2 between endometrial cancer and adjacent normal tissues. The Kaplan–Meier plotter online tool was employed to analyze the correlation between MTFR2 protein expression levels and overall survival as well as recurrence-free survival in endometrial cancer patients. The biological functions and associated pathways of MTFR2-related genes in endometrial cancer were analyzed using the GO and KEGG databases.

### 2.2 Tissue collection and immunohistochemistry

From August 2022 to March 2023, poorly differentiated (42 cases in total) and well differentiated (65 cases in total) endometrial cancer tissues diagnosed by surgery and pathology in the Third Affiliated Hospital of Zhengzhou University were collected (The patients’ age ranged from 35 to 65 years, with menarche occurring between 10 and 16 years, and menopause between 45 and 65 years. Their weight ranged from 45 kg to 75 kg, and the number of pregnancies was between 1 and 6, with the number of deliveries ranging from 0 to 4), as well as endometrial hyperplasia tissues (27 cases in total) diagnosed and curetted for abnormal bleeding in the same period. The inclusion criteria for the sample were that the patients had not previously undergone uterine surgery, radiotherapy, or chemotherapy, and cases with potential coexisting tumors or specific diseases were excluded. This study has been officially approved by the Medical Ethics Committee of the Third Affiliated Hospital of Zhengzhou University (Code of ethics documentation: 2021-076-01).

The paraffin tissue sections were baked for 1 h in advance, and then soaked in fresh xylene, 95% ethanol and 75% ethanol for several minutes, respectively, after baking, and washed and soaked with distilled water. Then, the antigen was repaired by heating in the sodium citrate buffer, and endogenous peroxidase was dripped. Afterward, the primary antibody of MTFR2 was incubated overnight at 4°C, and then the goat anti-rabbit IgG polymer labeled with enhanced enzyme was dripped. Finally, DAB color development, counterstaining, dehydration, transparency and film sealing were carried out. The staining results were evaluated and scored by two senior pathologists. To avoid experimental bias, we chose the blinded scoring method. The specific scoring criteria are as follows: the standard of immunohistochemistry is staining index, that is, positive reaction staining intensity × positive cell staining area; a score of 0–7 in the immunohistochemical staining index is considered as low expression, and a score of 8–12 is considered as high expression (16).

### 2.3 Cell culture

In this study, the immortalized human endometrial cell line hEM15A was provided by Thermos Biotechnology Co Ltd., and the human endometrial cancer cell lines HEC-1A, HEC-1B, KLE, and Ishikawa were purchased from Procell Biotechnology Co Ltd. Among them, HEM15A cells were cultured in DMEM/F12 medium, human endometrial carcinoma Ishikawa and KLE cells were cultured in DMEM medium, HEC-1A cells were cultured in McCoy’5A medium, and HEC-1B cells were cultured in MEM medium. The culture medium contains 10% serum and 1% double antibody, and the incubator conditions are 37°C and 5% CO2.

### 2.4 Cell transfection

Lentivirus transfection into HEC-1A cell line: An experimental group was established, and puromycin with various concentration gradients was used to determine the lowest effective concentration. Calculated the virus amount and added the virus solution and the proper amount of polybrene to the six-well plate to continue the culture. Replaced it with fresh medium after 12–16 h of culture, and screened the cells for about 10 days with selective medium containing antibiotics, so that the stably expressed cells could be preserved. Transfection efficiency test (qRT-PCR, Western blot) and subsequent cell function experiments were carried out after transfection.

Plasmid transfection of Ishikawa cell line: Add the transfection solution to the six-well plate, then replace with fresh complete medium for continuous culture. After transfection for 24 h, half of them were tested for transfection efficiency, and the last were used for subsequent cell function tests.

### 2.5 Quantitative real-time PCR analysis

Total RNAs were extracted from Trizol reagent (Cwbio, Beijing, China), and ReverTra Ace qPCR RT Master Mix (Takara Bio, Japan) was used to synthesize single-stranded cDNA. Finally, qRT-PCR reactions were performed using the PerfectStart Green qPCR SuperMix (Sevenbio). The primer sequences used areas follows:

GAPD, Forword, 5′-ATTCCACCCATGGCAAATTC-3′.
Reverse, 5′-GACTCCACGACGTACTCAGC-3′.
MTFR2, Forword, 5′-ATTTTGGCGTTCCTGTAGAACA-3′.
Reverse, 5′-CAGAGTTCAAGAGCGGGATCA-3′.

### 2.6 Western blot analysis

After extracting protein samples on ice with RIPAlysing agent (Beyotime Biotechnology), the concentration of protein samples was measured with BCA kit (Sevenbio). After SDS-polyacrylamide gel electrophoresis, the protein was transferred to polyvinylidene fluoride membrane. After that, incubated the primary antibody overnight at 4°C and hatched the secondary antibody for 1 h at room temperature. Finally, the electrogenerated chemiluminescence solution (A: B = 1:1) was applied to the membrane, followed by image processing using GelView 6000Plus after exposure.

### 2.7 CCK-8 assays

Cell suspensions were prepared and adjusted to appropriate (Generally, the cell concentration ranges from 1 × 10^4^ to 1 × 10^5^ cells/well) cell concentrations before being evenly seeded into 96-well plates with 5 replicate wells per experimental group. At 24 h, 48 h, 72 h, and 96 h, 10 μL of CCK-8 solution was added to each well, followed by continued light avoidance incubation for 2 h. Subsequently, the absorbance values (Optical Density, OD) of each well were measured. Charted the cell proliferation curve.

### 2.8 Clone formation assay

Cell suspensions were prepared and adjusted to appropriate cell concentrations before being evenly seeded into six-well plates. After a period of culture, the formation of cell colonies was observed under a microscope. The cells were fixed using 4% paraformaldehyde and subsequently stained with 1% crystal violet. Following natural drying, they were photographed and documented.

### 2.9 Transwell migration and invasion assay

Migration assay: Inoculated 200 μL of the mixed cell suspension (serum-free) into the upper chamber, and put the culture medium containing 15% FBS into the lower chamber about 24 h. Then, remove the non-migrating cells from the upper chamber, fix them with 4% paraformaldehyde, and stain with 1% crystal violet solution. Finally, wash with PBS, and capture images under a microscope for recording.

### 2.10 Invasion assay

Firstly, matrigel matrix glue was mixed into serum-free culture solution at 4°C in a ratio of 1:8, then spread in the upper chamber about 4 h until the colloid solidified. The remaining operation steps are the same as migration assay.

### 2.11 Wound healing assay

Drew two clear scratches with the gun head along the coordinate axis intersecting vertically and horizontally, and replaced it with serum-free medium to continue culture. At 0 h, 24 h, 48 h, and 72 h after the scratch was generated, the same position of the scratch was photographed under the microscope. At the later stage, the healing area of the scratch was quantitatively analyzed by Image J software.

### 2.12 Luciferase reporter assay

A luciferase expression reporter vector was used to insert a 3′-UTR segment of MTFR2 and its mutant. In order to validate the targeting of MTFR2 by miR-132-3p, endometrial cancer cells were co-transfected with the luciferase vector and a miR-132-3p mimic about 48 h, and measured the luciferase activity.

### 2.13 Subcutaneous tumorigenesis in nude mice

This study utilized female BALB/c nude mice aged between 6 and 8 weeks. Ubcutaneous xenograft models were established by transplanting control cells and endometrial cancer cells experimentally. Tumor volumes were measured at 7-day intervals. After a duration of 4 weeks, the mice were euthanized, and tumor weights were assessed. The animal research protocol received approval from the Animal Care and Use Committee of Zhengzhou University (Code of ethics documentation: 2024-038-01) and adhered to the Animal Care Guidelines of Zhengzhou University.

### 2.14 Statistical analysis

All bioinformatics analyses were performed by R software (Version 3.4; R Foundation for Statistical Computing, Vienna, Austria). All Statistical analyses were performed with SPSS 26.0 for Windows (SPSS Inc., Chicago, IL, United States). Chi-square test and Fisher Chi-square were used to compare the Clinic-pathologic factors and the *t* test or one-way ANOVA analysis were conducted to compare continuous variables, the Log-rank test and Kaplan–Meier curves were involved to survival analysis. The relationship between MTFR2 expression and clinicopathological features of patients was assessed using the chi-square test. All the statistics associated with clinical specimens were processed using Prism 9.0 (GraphPad Software Inc., La Jolla, United States). Statistical significance was set to the probability values of *p* < 0.05.

## 3 Results

### 3.1 MTFR2 is upregulated in endometrial carcinoma and related to its progression

**Figure 1:**
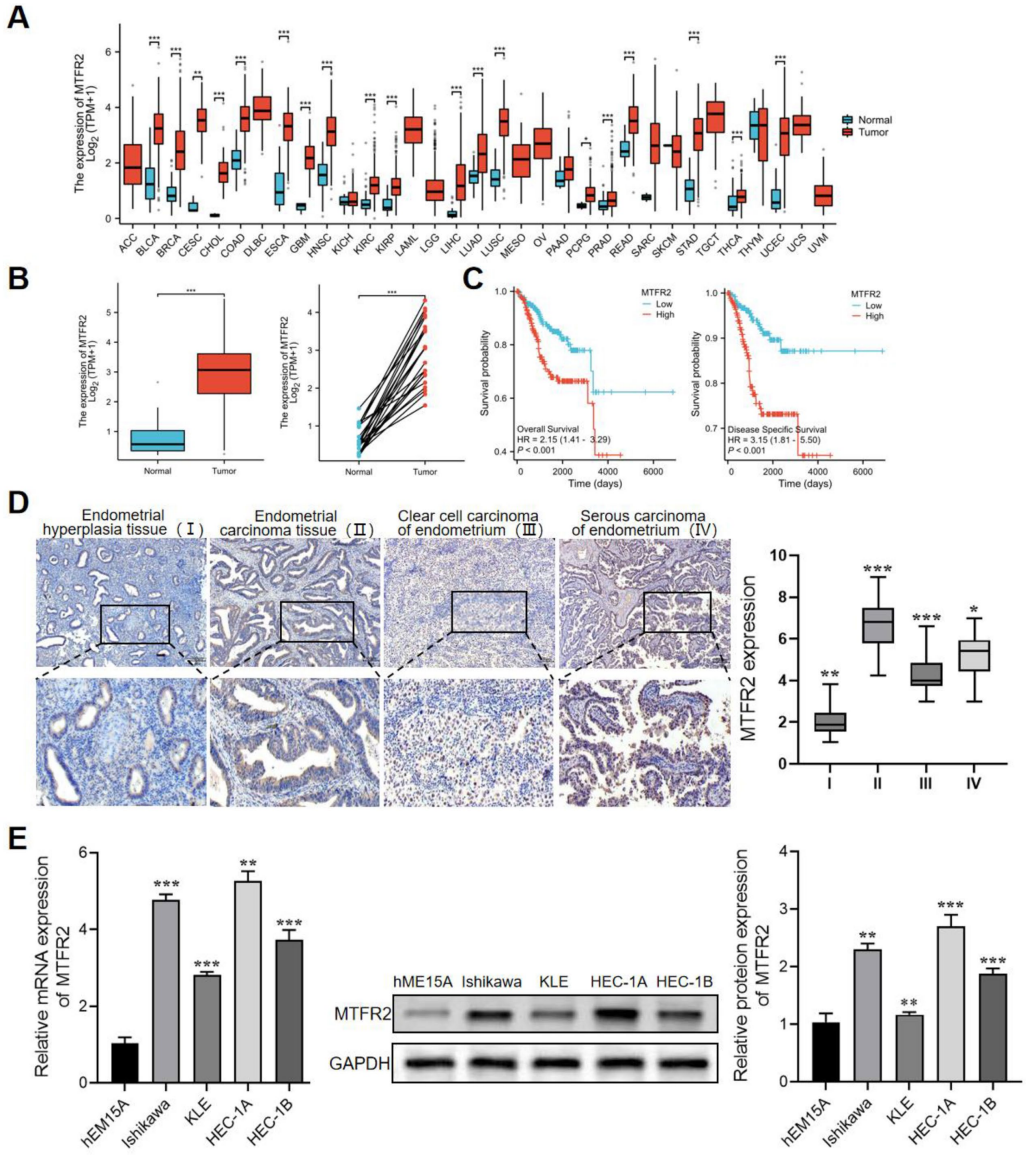
MTFR2 is highly expressed in endometrial carcinoma and is a potential prognostic marker. **(A)** Differential expression of MTFR2 in pan-cancer. **(B)** In TCGA database, the expression of MTFR2 was analyzed in 554 cases of endometrial carcinoma and 35 cases of normal endometrium. **(C)** Correlation analysis of endometrial cancer patients with overall survival and relapse-free survival based on TCGA database. **(D)** Immunohistochemical analysis of MTFR2 expression in endometrial hyperplasia, endometrial adenocarcinoma, clear cell carcinoma and serous carcinoma. Scale: 100 mm. **(E)** The differential expression of MTFR2 between endometrial carcinoma cell line and human endometrial immortalized cell line was detected by qRT-PCR and Western blot. GAPDH was used as the standard control. All the data are presented in the form of mean ± SD from three independently performed experiments (***p < 0.001, **p < 0.01, *p < 0.05).

As shown in Figure 1A, compared with normal tissues, the expression of MTFR2 is upregulated, such as cervical cancer, renal clear cell carcinoma, lung adenocarcinoma, colon cancer, and other tumors. In TCGA, MTFR2 was significantly overexpressed in endometrial carcinoma tissues, whether compared with paired or unpaired normal endometrial tissues (Figure 1B). In addition, Kaplan–Meier plotter analysis showed that the overall survival rate and recurrence-free survival rate of endometrial cancer patients with high expression level of TRIP13 were significantly lower than those with low expression level (Figure 1C). Finally, IHC staining further confirmed that MTFR2 was highly expressed in endometrial adenocarcinoma, serous carcinoma and clear cell carcinoma (Figure 1D). Generally speaking, the above results show that MTFR2 is highly expressed in endometrial carcinoma, which may be one of the poor prognosis factors of endometrial carcinoma. We evaluated the expression level of MTFR2 in various endometrial carcinoma cell lines, as depicted in Figure 1E. The expression of MTFR2 was significantly higher in HEC-1A, HEC-1B, KLE, and Ishikawa cell lines compared to the hEM15A cell line. Among these four endometrial cancer cell lines, HEC-1A exhibited relatively high TRIP13 expression, while Ishikawa showed relatively low TRIP13 expression (Figure 1E).

### 3.2 MTFR2 promotes the proliferation, migration, and invasion of endometrial cancer cells

**Figure 2:**
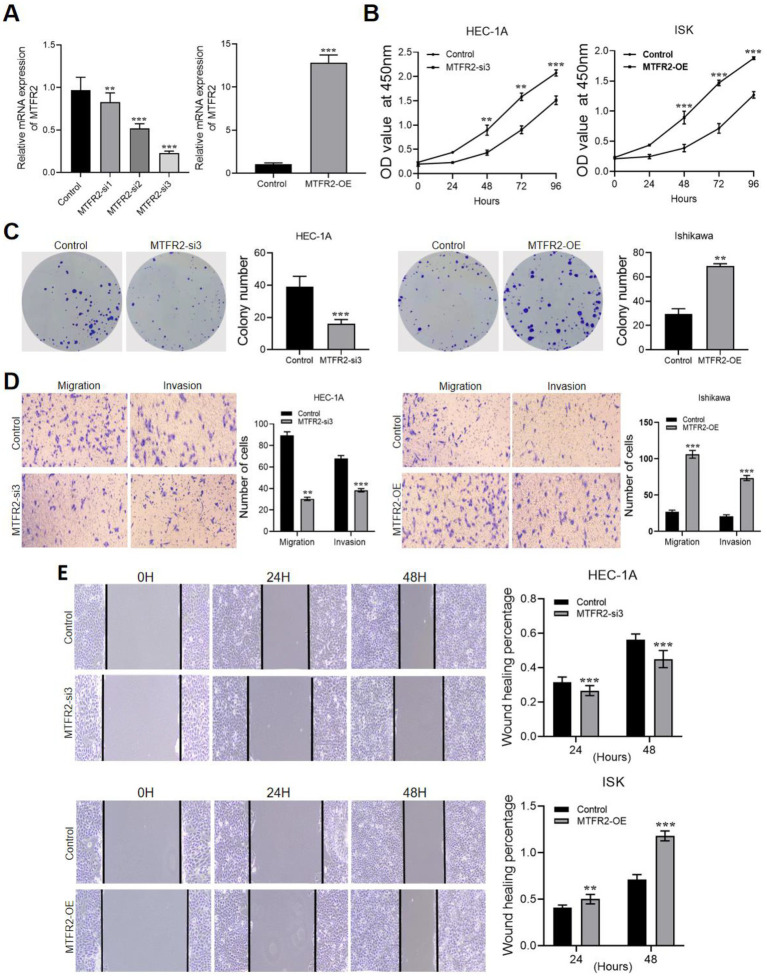
MTFR2 enhances the proliferation, migration and invasion of endometrial cancer cells. **(A)** Efficiency verification of HEC-1A cell line to construct MTFR2-si3 stable cell line. Efficiency verification of Ishikawa cell line in constructing MTFR2-OE stable cell line. **(B)** CCK-8 experiment was used to detect the changes of proliferation ability of endometrial cancer cells after MTFR2 was knocked down or overexpressed. **(C)** The colony-forming ability of endometrial cancer cells was detected by cell colony-forming experiment after MTFR2 was knocked down or overexpressed. **(D)** Transwell Asset detected the changes of migration and invasion of endometrial cancer cells after MTFR2 knock-down or overexpression. **(E)** Cell scratch test was used to detect the changes of migration ability of endometrial cancer cells after MTFR2 knock-down or overexpression. All the data are presented in the form of mean ± SD from three independently performed experiments (***p < 0.001, **p < 0.01).

To investigate the biological function of MTFR2, we established a knockdown cell line by lentivirus transduction and overexpressed MTFR2 in the Ishikawa cell line using a lentivirus system. The results of qRT-PCR expressed that the knockdown effect of MTFR2-si3 is the strongest, which is presented in Figure 2A. The CCK-8 and cell colony formation results showed that MTFR2-si3 significantly reduced the proliferation ability of HEC-1A cells (Figure 2B), while overexpression of MTFR2 significantly enhanced the proliferation ability of Ishikawa cells (Figure 2C). The findings demonstrated that MTFR2 knockdown significantly inhibited the growth of HEC-1A cells, whereas MTFR2 overexpression promoted proliferation in Ishikawa cells. Moreover, scratch and Transwell assays were conducted to examine cell migration and invasion, respectively (Figures 2D,E). The results revealed that MTFR2 knockdown inhibited migration and invasion, whereas MTFR2 overexpression enhanced cell migration and invasion.

### 3.3 MTFR2 enhances endometrial cancer cell tumorigenesis *in vivo*

**Figure 3:**
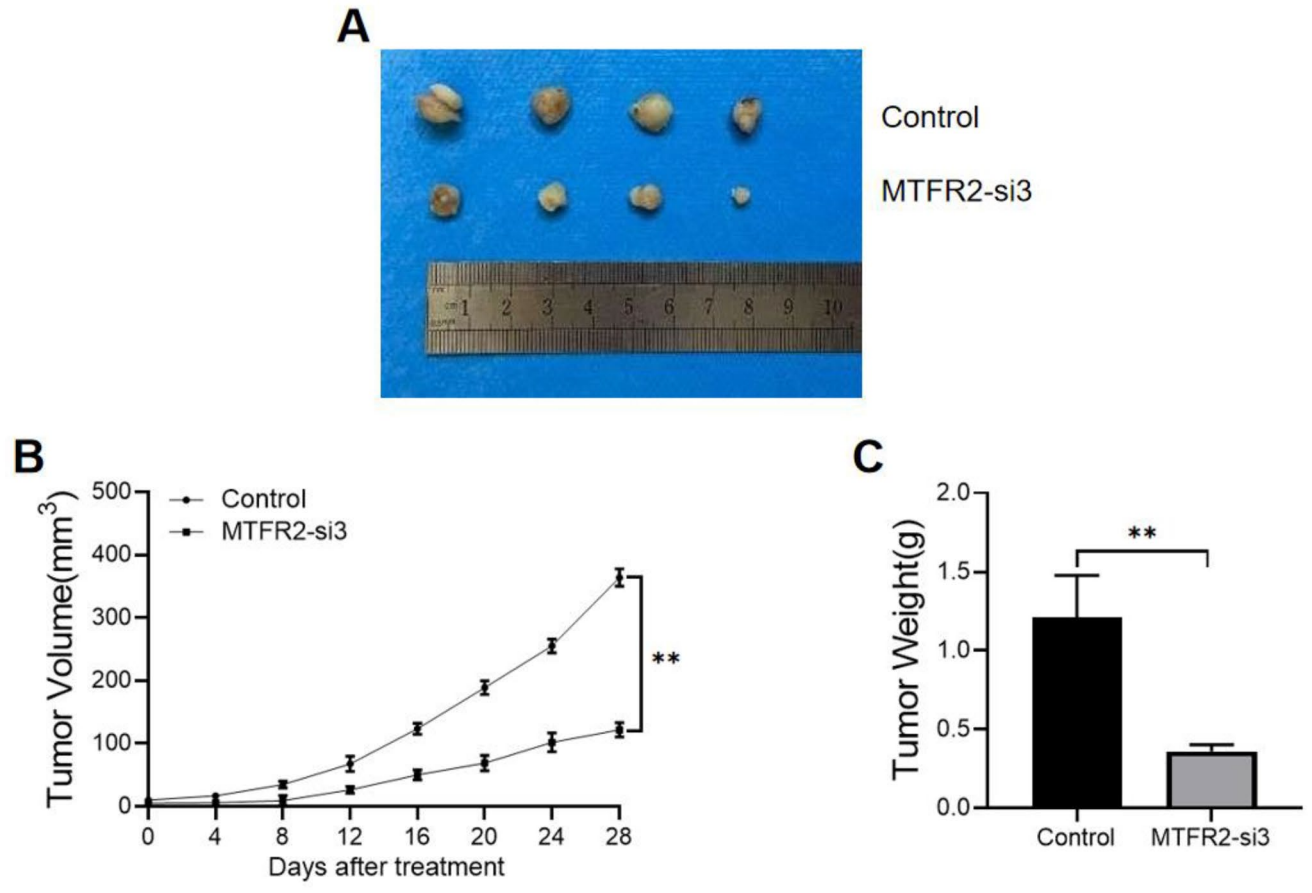
MTFR2 enhances endometrial cancer cell tumorigenesis *in vivo*. **(A)** Representative images of subcutaneous xenograft tumors of MTFR2-si3 and control groups in nude mice. **(B)** The growth curve of the xenograft tumors originating from MTFR2-si3 and control groups. **(C)** The weight of xenograft tumors for the MTFR2-si3 and control groups. All the data are presented in the form of mean ± SD from three independently performed experiments (**p < 0.01).

To further confirm the role of MTFR2 in promoting endometrial cancer oncogenesis, we established a xenograft model by subcutaneously injecting MTFR2-silenced HEC-1A cells into mice. Interestingly, the MTFR2-si3 group exhibited smaller tumors compared to the control group (Figure 3A). Moreover, the MTFR2-si3 group showed a significantly slower rate of tumor growth compared to the control group (Figure 3B). Additionally, the tumor weight in the MTFR2-si3 group was significantly lower than that in the control group (Figure 3C). These findings collectively indicate that silencing MTFR2 suppressed the growth of endometrial cancer cells in the animal model.

### 3.4 miR-132-3p directly targets MTFR2

**Figure 4:**
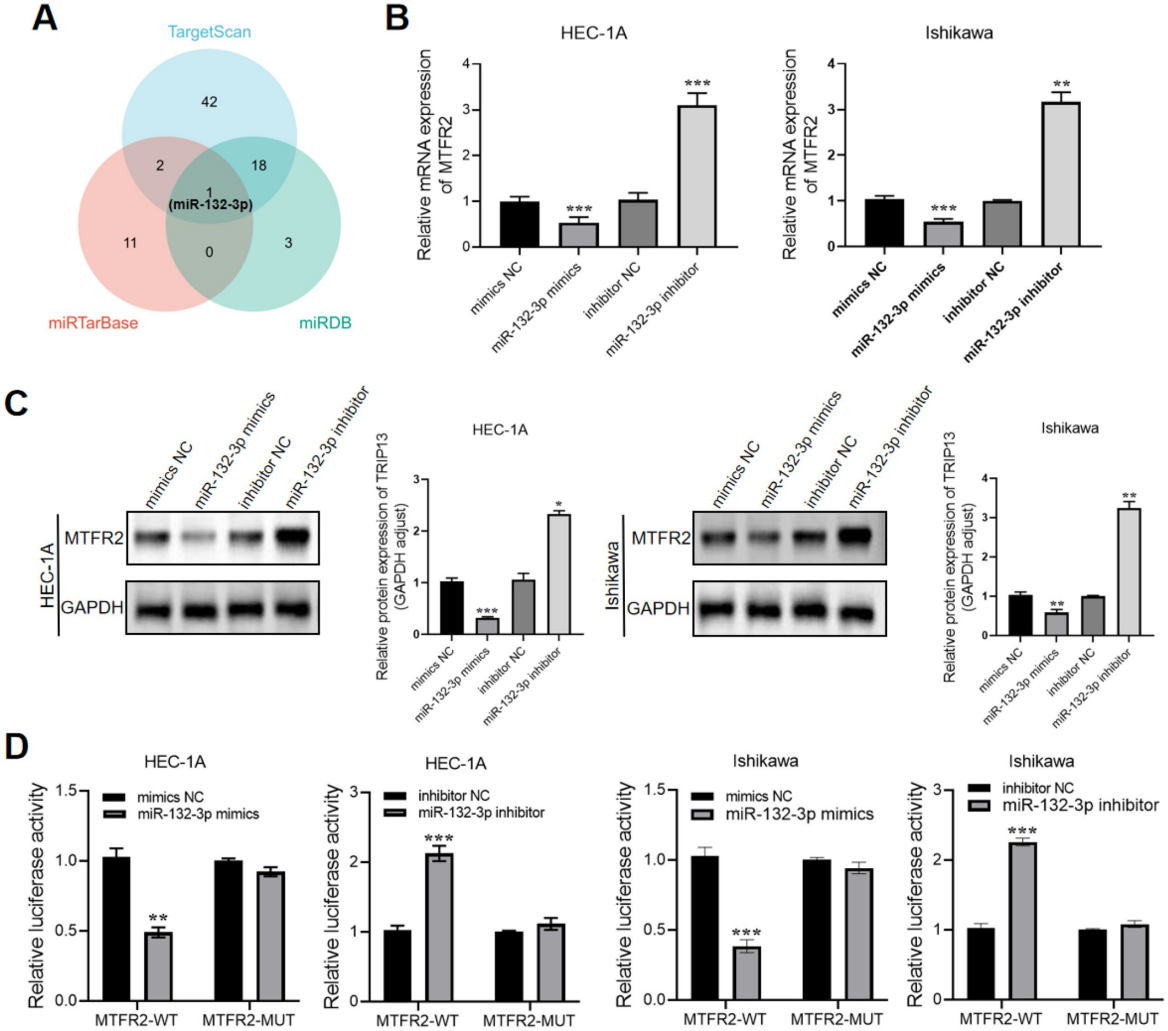
MTFR2 is a direct target of miR-132-3p. **(A)** Venn diagram showing the overlap microRNAs from four microRNA prediction algorithms. **(B)** qRT-PCR analysis of MTFR2 mRNA expression levels in HEC-1A and Ishikawa cells treated with miRNA-132-3p mimics or inhibitors. **(C)** Western blotting analysis of MTFR2 protein levels in HEC-1A and Ishikawa cells treated with miRNA-132-3p mimics or inhibitors. **(D)** Luciferase activity assays showing the direct binding efficiency of miR-132-3p and its putative MTFR2 3′-UTR targets. Data are presented as the mean ± SD of three independent experiments. (*p < 0.05, **p < 0.01, ***p < 0.001).

MicroRNAs (miRNAs) play crucial roles in the development and progression of cancer. Based on the KEGG analysis results shown in Figure 2B, we investigated whether dysregulated miRNA levels could affect the expression of MTFR2. To address this question, we utilized TargetScan, miRTarBase, and miRDB databases to identify miRNAs that potentially target MTFR2. As a result, we identified one candidate miRNA (Figure 4A). Subsequently, we examined the functional roles of miR-132-3p by transfecting miR-132-3p mimics into HEC-1A cells and miR-132-3p inhibitors into Ishikawa cells. Notably, miR-132-3p showed strong suppression of MTFR2 mRNA and protein levels (Figures 4B,C). To confirm whether miR-132-3p directly targets MTFR2, luciferase experiments were conducted using the MTFR2 3′-UTR linked to the luciferase coding region. A significant decrease in luciferase activity was observed when comparing the miR-132-3p mimic group to the control group (Figure 4D). Overall, our results provide evidence supporting the direct binding of miR-132-3p to the 3′-UTR of MTFR2, leading to the downregulation of MTFR2 translation.

### 3.5 MTFR2 activates PI3K/Akt signaling pathway during endometrial cancer progression

**Figure 5:**
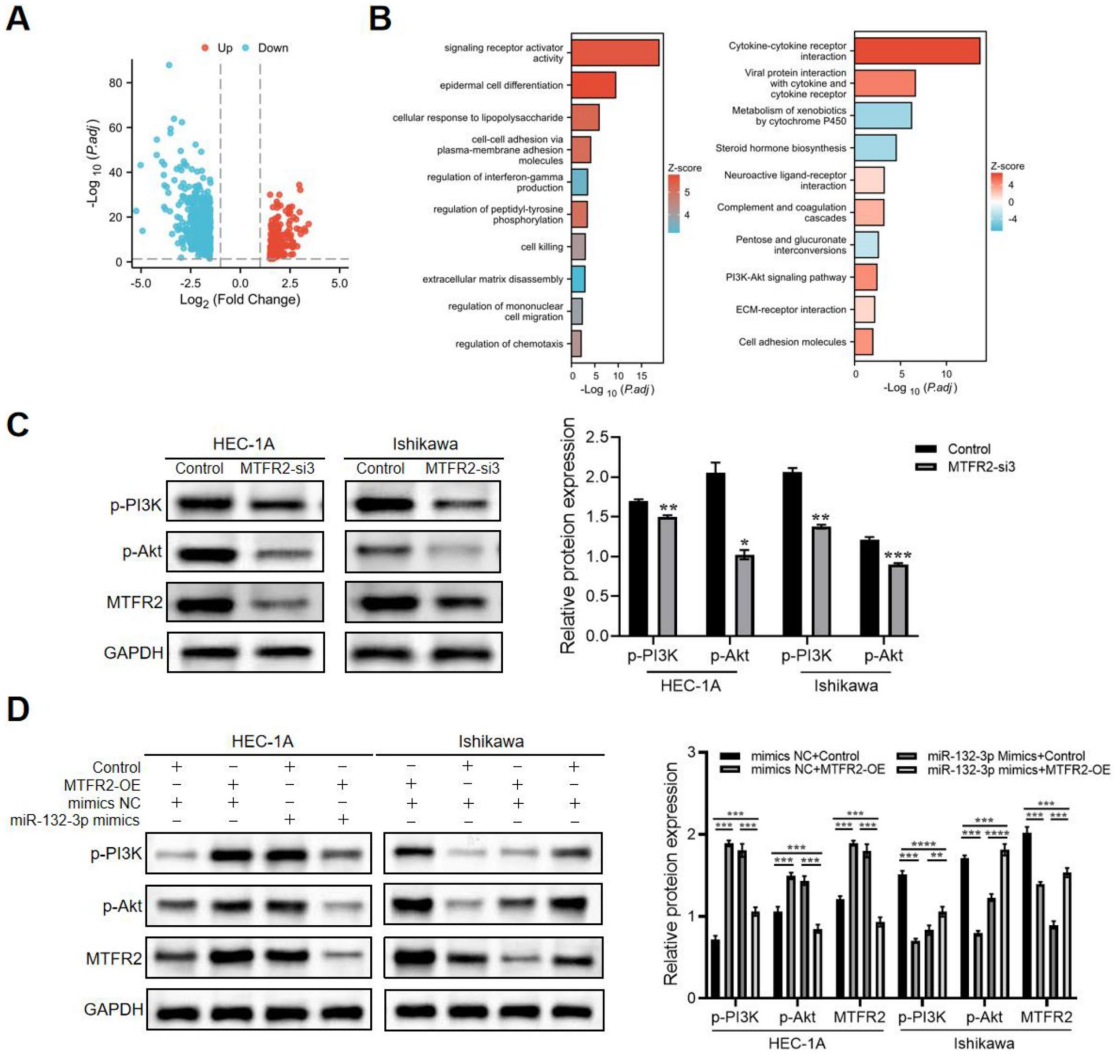
MTFR2 activates PI3K/Akt signaling pathway during endometrial cancer progression. **(A)** Volcano plot showing the differentially expressed genes (DEGs) between the high and low MTFR2 expression groups of patients with endometrial cancer. **(B)** Results of Gene ontology (GO) analysis and KEGG pathway enrichment analysis of DEGs between the high and low MTFR2 expression groups of patients with endometrial cancer. **(C)** HEC-1A and Ishikawa cells were transfected with si-RNAs or si-NC. The levels of p-PI3K and p-Akt were assessed using western blot analysis. **(D)** MTFR2-overexpressing cells or corresponding control cells underwent transfection with miR-132-3p mimics or mimics NC. p-PI3K, p-Akt, and MTFR2 expression identified by western blot. GAPDH served as a control. Data are presented as the mean ± SD of three independent experiments. (*p < 0.05, **p < 0.01, ***p < 0.001, ****p < 0.0001).

The results of the KEGG analysis revealed a significant enrichment of the PI3K/Akt signaling pathway in MTFR2-upregulated cases (Figures 5A,B), suggesting its potential role as a key contributor to endometrial cancer progression. To validate this hypothesis, we conducted Western blotting and observed that the phosphorylation of p-PI3K and p-Akt was suppressed upon MTFR2 knockdown (Figure 5C). Conversely, MTFR2 overexpression in Ishikawa cells resulted in a marked increase in p-PI3K and p-Akt levels, which were attenuated by transfection with miR-132-3p mimics (Figure 5D). These findings collectively demonstrate that MTFR2 regulates the growth of endometrial cells by modulating the PI3K/Akt signaling pathway.

## 4 Discussion

Further investigation into the functions of MTFR2 in endometrial cancer is warranted, considering its potential involvement in the pathogenesis and progression of various tumor types. In this study, we observed elevated levels of MTFR2 in endometrial cancer, which were associated with unfavorable clinical outcomes. Moreover, we elucidated the role of MTFR2 in promoting endometrial cancer tumorigenesis through the activation of the PI3K/Akt signaling pathway. Additionally, we identified a direct interaction between MTFR2 and miR-132-3p, suggesting a potential regulatory mechanism for the oncogenic effects of MTFR2.

Mitochondrial fission regulator 2 is a vital mitochondrial protein involved in the regulation of mitochondrial dynamics and function. Recent investigations have demonstrated increased expression of MTFR2 in cancer cells, which has been associated with unfavorable prognosis (18, 19). In our study, we corroborated the up-regulation of MTFR2 expression in endometrial carcinoma through analysis of the TCGA database. Furthermore, PCR and WB experiments confirmed the significant up-regulation of MTFR2 expression in five endometrial carcinoma cell lines. Immunohistochemical analysis revealed elevated MTFR2 expression in various types of endometrial carcinoma tissues. Notably, higher MTFR2 expression was significantly correlated with reduced overall survival probability and relapse-free survival, indicating the potential of MTFR2 as a prognostic marker for endometrial cancer studies. Multiple lines of evidence have linked MTFR2 overexpression to cancer initiation and progression. For instance, a previous study reported that a prognostic signature comprising 10 mitochondrial dynamics genes, including MTFR2, played crucial roles in mitochondrial fission and had the potential to serve as important predictors and therapeutic targets for hepatocellular carcinoma (20). Additionally, another study demonstrated that HOXC10 overexpression activated MTFR2 expression, thereby enhancing the proliferation, clone formation, invasion, and migration of colorectal cancer cells (21). Our present study further supports the notion that MTFR2 plays critical roles in promoting endometrial cancer cell growth by facilitating cell cycle progression and suppressing apoptosis. *In vitro* experiments further demonstrated that MTFR2 enhanced tumorigenesis in cells. Collectively, our findings underscore the significance of MTFR2 as a key contributor to endometrial tumorigenesis.

Dysregulated expression of microRNAs has been found in various types of cancer, including endometrial cancer (22–24). Our KEGG enrichment analysis revealed a significant correlation between elevated MTFR2 expression and microRNAs in cancer. Typically, microRNAs facilitate the degradation of target mRNA, thereby inhibiting translation. Through comprehensive bioinformatic analysis and experimental validation, we confirmed that miR-132-3p directly targets MTFR2 by binding to its 3′-UTR. Notably, overexpression of miR-132-3p led to significant downregulation of MTFR2 in endometrial cancer tissues.

Subsequent experiments yielded results indicating that the overexpression of miR-132-3p could partially suppress the oncogenic function of MTFR2, thereby further confirming the direct targeting of MTFR2 by miR-132-3p. In a similar vein, another study suggested that heat stimulation promotes the progression of squamous cell carcinoma, and miR-132-3p mediates this process by directly targeting KCNK2 (25). Furthermore, a separate investigation reported that miR-132-3p regulates antibody-mediated complement-dependent cytotoxicity in colon cancer cells by directly targeting CD55 (26). Collectively, these findings provide compelling evidence supporting the negative regulatory role of miR-132-3p in the oncogenic activity of MTFR2 in endometrial cancer. Moreover, based on our study, it can be inferred that miR-132-3p could also be considered a potential prognostic biomarker. Recent studies have also highlighted that, because it can be analyzed in circulating blood, this analysis minimizes invasiveness for patients (27, 28).

To gain further insights into the molecular mechanism by which MTFR2 influences endometrial cancer progression, we conducted bioinformatic analysis. Interestingly, we observed a significant correlation between elevated MTFR2 levels and the activation of the PI3K/Akt signaling pathway. Additionally, there-expression of miR-132-3p partially attenuated Akt phosphorylation in endometrial cancer. Consistently, previous research has demonstrated that the PI3K/Akt pathway can induce the epithelial-mesenchymal transition (EMT) process by downregulating epithelial markers and upregulating mesenchymal markers and EMT-specific transcription factors, thereby promoting metastasis in colorectal carcinoma (29). However, the precise regulatory mechanism underlying MTFR2-mediated activation of the PI3K/Akt pathway remains incompletely understood. A separate study reported that MTFR2 promotes oral squamous carcinoma by shifting oxidative phosphorylation (OXPHOS) to glycolysis. These studies collectively suggest that OXPHOS could represent a potential target through which MTFR2 promotes the progression of endometrial cancer. However, further experimental validation is needed to determine whether MTFR2 activates the PI3K/Akt signaling pathway through IGF2 or IGF1R.

## 5 Conclusion

In summary, our study highlights the significant upregulation of MTFR2 in endometrial cancer, which leads to the activation of the PI3K/Akt signaling pathway and plays a crucial role in promoting tumor cell proliferation. This aberrant upregulation of MTFR2 is partially attributed to posttranscriptional regulation by miRNA-132-3p. Finally, our data establish MTFR2 and miRNA-132-3p as a novel and promising prognostic biomarker for patients with endometrial cancer. The findings of this study emphasize the importance of MTFR2 in the development of endometrial cancer and suggest its potential as a therapeutic target.

## Data Availability

Publicly available datasets were analyzed in this study. This data can be found in the article. The original contributions presented in the study are included in the article, further inquiries can be directed to the corresponding author. All data will be shared upon reasonable request.
